# Closing the Mentorship Gap: Implementation of Speed Mentoring Events for Women Faculty and Trainees in Anesthesiology

**DOI:** 10.1089/whr.2020.0095

**Published:** 2021-02-16

**Authors:** E. Morgan Pollard, Emily E. Sharpe, Bhargavi Gali, Susan M. Moeschler

**Affiliations:** Department of Anesthesiology and Perioperative Medicine, Mayo Clinic, Rochester, Minnesota, USA.

**Keywords:** mentorship, sponsorship, gender, diversity, academic medicine, speed mentoring

## Abstract

***Introduction:*** Mentorship is a key component to success in academic medicine. Women are under-represented in leadership positions within medicine. Women are less likely to identify mentors than men. Speed mentoring is an innovative strategy to facilitate mentorship in academic medicine.

***Materials and Methods:*** A speed mentoring event for women faculty members in an academic anesthesiology department was held, followed by a second event for trainees. Attendees completed surveys about mentorship experiences at baseline and in follow-up. Questions were rated on a 7-point Likert scale with 1 = strongly disagree and 7 = strongly agree with values reported as median (1st, 3rd quartile).

***Results:*** Baseline surveys indicated poor satisfaction with mentoring in the prior 6 months as 4.5 (3, 5.25). Twelve months later, mentees reported increased satisfaction with mentoring 6 (6, 6). Mentors and mentees felt their time was well spent during both events. There was an increase in the number of mentors identified after the events by both groups.

***Conclusions:*** Our results suggest speed mentoring is well received and impactful with minimal time and monetary investment. The attendees of the events identified an increased number of mentors after speed mentoring events, and this effect was maintained at 6–12 months. Speed mentoring may be one path to providing support for women to advance their careers in academic medicine. More research is warranted to better evaluate effectiveness of formats such as speed mentoring to facilitate improved mentorship for women in academic anesthesiology.

## Introduction

Women continue to be under-represented in academic medicine, comprising only 39% of full-time faculty, 22% of full professors, and 16% of departmental chairs and medical school deans.^1,2^ Only 22.7% of invited speakers for academic grand rounds were women.^3^ At prestigious medical journals, only 10% of senior authors and 7% of editors-in-chief are women.^2^

The under-representation of women at higher ranks in academia may result, in part, in a lack of mentorship in comparison with their male colleagues. Mentorship has been identified as a key component of success in business as well as in academic medicine.^4^ Mentorship is associated with increased faculty retention, productivity, and promotion.^4^ However, women medical students, residents, and junior faculty were less likely to identify a current or past mentor than their male colleagues.^4^

Efforts to address these discrepancies should include improvement in recruitment, retention, and mentorship to ensure career satisfaction.^5^ Initiating a mentoring relationship may be difficult, particularly for junior faculty and trainees. A previous research survey completed among faculty within our department found that only 30% of respondents identified a mentor. Thus, speed mentoring events were scheduled to encourage multiple mentor sessions that would hopefully lead to further mentor–mentee relationships to enhance work satisfaction and retain and advance women faculty in their careers.

## Materials and Methods

This study was deemed exempt by the Mayo Clinic Institutional Review Board. The gap in mentorship in our department led a group of women faculty to create a formal event to foster mentor–mentee relationships among women. Speed mentoring was selected as the format for our department's event since it allows multiple high-yield interactions in a short period of time. This approach was originally described by Cook et al.^6^ and is an event that creates multiple pairings of mentees with mentors in a structured environment.

The aim of the intervention was to engage in discussion of mentee-identified goals, including clinical practice, research, education, and work–life integration. We also sought to increase interdepartmental satisfaction with mentorship and to facilitate sponsorship.

The pilot intervention was open to all women who were junior faculty (academic rank of instructor or assistant professor) in the Department of Anesthesiology in Rochester, Minnesota, and was held on a weekday evening in May 2018. Participants were invited *via* e-mail 6 weeks before the event. Six women faculty, with leadership and research experience were invited to serve as mentors and nine junior faculty attended as mentees.

The event lasted 2 hours and 30 minutes, during which every mentee met with three mentors for 30 minutes each. Each mentee was given a schedule with color-coded time slots to speak with mentors. The mentor–mentee encounters were not structured, but mentees were encouraged to prepare questions before the event. The invitation e-mail stated “in preparation to the event, we ask you to reflect on 2–3 questions/goals for the mentors at the event. These can be broad or very specific, and will help to create the pairing grid for the event.”

Each participant completed baseline surveys on paper before and after the speed mentoring event. A seven-question follow-up survey was input into REDCap and a link was e-mailed to all participants at 1, 3, 6, and 12 months (Supplementary Fig. S1).

The trainee event was organized in a similar manner and was held in December 2018. All residents, fellows, and junior faculty in our department were invited *via* e-mail to attend. Six senior faculty mentors and 12 (mentees) residents, fellows, and junior faculty attended the event. Each mentor and mentee was given a schedule with four time slots to meet for 30 minutes with a different senior faculty member.

All attendees completed baseline surveys before and after the event in person on paper. Follow-up surveys were collected at 1, 3, and 6 months *via* e-mail links to REDCap. Twelve months follow-up was not conducted due to several trainees completing training and relocating. Questions on surveys were rated on a 7-point Likert scale with 1 = strongly disagree, 4 = neutral, and 7 = strongly agree. All values are reported as median (1st, 3rd quartile). Data for the faculty and trainee events were combined. Themes for the free responses were categorized by author consensus and not analyzed further.

## Results

Twenty-one mentees and 12 mentors participated in the events. Three mentors participated in both events. The mentees comprised 11 junior faculty (52%), 6 residents (29%), and 4 fellows (19%). Eighteen mentees were women and three mentees were men. Baseline surveys revealed participants desired mentorship in the following areas: clinical practice = 6 (6, 7), research = 7 (6, 7), academic advancement = 7 (6.75, 7), and work–life integration = 7 (7, 7). The mentees were asked to rate their satisfaction with mentoring at baseline and on follow-up surveys and the results are reported in Table 1.

**Table 1.** Perceptions of Mentoring Quality at Baseline and During Follow-Up After Speed Mentoring Events

	Baseline (*n* = 21)	1 month (*n* = 19)	3 months (*n* = 14)	6 months (*n* = 10)	12 months (*n* = 5)
I am satisfied with the mentoring I have received in the past 6 months (baseline), or since the speed mentoring event.^a^	4.5 (3, 5.25)	6 (5.5, 6)	6 (6, 6)	6 (6, 6)	6 (6, 6)
I would benefit from more intensive mentoring.^a^	—	6 (6, 7)	6 (5, 6.75)	5.5 (5, 6.75)	6 (5, 7)
I have been successful in finding mentors as I need them.^a^	4 (3, 5)	6 (5, 6)	5.5 (5, 6)	5.5 (5, 6)	6 (6, 6)

Values reported as median (1st, 3rd quartile).

^a^ 7-point Likert scale (1 = strongly disagree, 2 = disagree, 3 = slightly disagree, 4 = neutral, 5 = slightly agree, 6 = agree, and 7 = strongly agree).

**FIG. 1.**
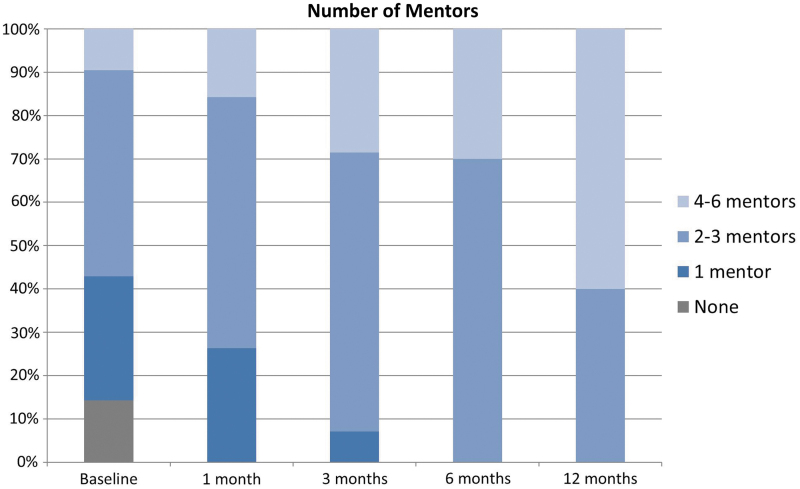
Number of mentors reported by mentees over time.

The number of mentors for each mentee increased after the speed mentoring event, and remained stable for the follow-up period (Fig. 1). At baseline, 3/21 (14%) and 6/21 (29%) identified 0 and 1 mentors, respectively. At 12 months, all mentees who responded were able to identify at least two mentors.

All participants felt their time was well spent during the event [mentees = 7 (7, 7); mentors = 7 (7, 7)]. In addition, all participants indicated they would slightly agree, agree, or strongly agree with recommending a similar event to a colleague.

Surveys included space for free text comments to garner opinions about the utility of the events, interactions with mentors, and suggestions for improvement. Mentees found the speed mentoring event to be useful and shared that it provided an opportunity to establish connections and relationships with senior leaders in the department. In addition, participants thought encouraging formal follow-up would be beneficial. A representative sample of comments has been summarized in Table 2.

**Table 2.** Representative Quotes from Open-Ended Responses

	Women faculty event	Trainee event
Utility of mentoring event	“Extremely useful. Each one of (the mentors) provided different insight, background, and experience to help answer my questions.” “… Jump-started mentoring relationships that I needed.” “Great to sit down with people who I admire in the practice and hear their insight and feedback.” “… Came up with an action plan for prioritizing writing my manuscripts… I've (already) completed one and started working on the next one.” “I've sought out national committee positions and presentations.”	“Very useful. An opportunity to talk with others you may not organically interact with.” “… It gave me inspiration and ideas for goal setting and research progress.” “Very useful for sparking conversation on current projects and ideas and providing the impetus for further work.”
Interaction with mentors	“Gave me face-to-face time with a mentor who works at a different campus, which was great.” “… enjoyable/rejuvenating experience to feel like part of a larger supportive group.” “… Validating to hear from mentors that the project I was working on was worthwhile.” “I feel that I have established a relationship with (the mentors) now and can approach them more easily.”	“I get the impression that my speed mentors would be willing to offer more mentoring and advice if I were to seek it out” “… [Interacting with the mentors] left the door open for future interactions and opportunities for development.” “Broke down barriers as it relates to approachability of leaders within the department.”
Ideas for improvement	“This is a great event that we should offer to other members of the department.” “Coming prepared with focused questions was essential to the format…” “Maybe set up a scheduled follow-up… and go over what the mentee has done.”	“… Aligning fields of interest with mentees might also be useful as the event grows, to increase the odds of common interests.” “… more useful the earlier in training.” “Provide a guided list of questions to help efficiently direct the session.” “Consider hosting the event earlier in the day… by the end, [participants were] understandably quite fatigued.” “…formal follow-up could be helpful with some of the mentors/mentees…”

## Discussion

Reviews of mentoring studies have found that mentorship programs for women can lead to increased success in academic medicine, although there is minimal literature on speed mentoring outcomes.^1,7^ Various mentoring modalities have reported improvements in academic and career development in addition to personal satisfaction.^7^ These have predominately involved dyadic, peer, and group mentorship models.

The first speed mentoring event in academic medicine was published by Cook et al.^6^ They reported high event satisfaction from both mentors and mentees, yet satisfaction with mentoring did not change 12 months after the event, from a mean score of 4.7 at baseline on a 7-point scale to a mean of 4.6 at 12 months.^6^ In comparison, mentees in our study indicated increased satisfaction with mentorship after 12 months, from a baseline median of 4.5 (on a similar 7-point scale) to a median of 6.

Speed mentoring is a low-resource modality that may provide increased opportunities for development of mentoring relationships. As there are less women who advance in academic careers, this may allow for the ability to provide mentorship for multiple women with limited need for longer time and resource needs. Our events were viewed as an efficient use of time and all participants indicated they would recommend a similar event to a colleague.

Lack of mentorship has been cited as a reason for the gap in academic achievement between women and men.^2^ In addition, mentorship has been associated with career satisfaction, and may help increase retention of women faculty in academic departments.^8^ Implementation of a minimal resource event such as speed mentoring may allow for early identification of possible mentors for women to address bias and improve retention and academic progress.

Mentorship can be beneficial for both the mentor and mentee, with the primary focus on the development of the mentee. Recently, it has been suggested that mentorship may not be enough to level the playing field for women.^9^

Sponsorship (active support by someone in the organization with significant influence)^10^ can help junior talent advance while also expanding the impact of senior leadership, but only when both parties stand to benefit.^11^ Women in business “are likely to be over-mentored and under-sponsored,” and the literature thus far suggests the same phenomenon exists in medicine.^12^ One possible hypothesis is that women mentees may have less powerful mentors who are, therefore, unable to act as sponsors.^13^

Developing mentorship relationships may be the first step along the continuum of professional engagement that can lead to sponsorship and career advancement. Hopefully, working on systemic leveling of the mentorship and sponsorship gap will also help alleviate some of the well-documented gender disparities in leadership positions and pay.^11^

The findings from our department's initial speed mentoring interventions have several limitations. The sample sizes are small and potentially biased. There were many mentees who did not complete follow-up during the study and 12-month follow-up was not offered to attendees of the trainee event. Mentors and mentees who participated all responded by e-mail, implying they are motivated to invest their time and efforts into mentorship at baseline. We had no control group; therefore, the junior faculty and trainees may have found more mentors over time on their own without the intervention of a speed mentoring event. Furthermore, detailed information about mentoring relationships or outcomes such as collaborations or projects were not collected.

The strengths of our interventions include high levels of reported satisfaction with the events and the low cost and small time commitment required of all participants for the event. The successes of our initial speed mentoring events have resulted in further events within our department.

Future research is needed to determine optimal interventions to improve mentorship and sponsorship for women anesthesiologists in all career stages. Future interventions should include a larger number of mentees as well as a control group to better evaluate the intervention. Once more data are known regarding when and how to best intervene, training programs and academic departments should endorse successful interventions to improve access of mentorship to all individuals seeking such relationships.

## Conclusions

In conclusion, speed mentoring is a low-cost intervention requiring minimal time commitment for both mentees and mentors. After two speed mentoring events, our survey data suggest high event satisfaction and increased satisfaction with mentoring. Many academic medicine departments and academic training programs promote mentorship through assigned mentors. The positive results of our intervention suggest this could be an additional tool to facilitate compatible mentor–mentee relationships. In addition, speed mentoring provides an opportunity for junior faculty and trainees to seek out mentorship from senior faculty members.

## Supplementary Material

Supplemental data
