# Supplementary material for: Closing the Mentorship Gap: Implementation of Speed Mentoring Events for Women Faculty and Trainees in Anesthesiology
Source: Womens Health Rep (New Rochelle). 2021 Feb 16;2(1):32–6. doi: 10.1089/whr.2020.0095 (PMC7935037; doi:10.1089/whr.2020.0095)
Supplement: Supplemental data [file Supp_Fig1.pdf]

STUDY ID: (DOB: Month-Day)
Gender:
Age:
Academic Rank:

# Mentoring Event: Baseline

1. How many mentors do you currently have?

a) None　　b) 1　　c) 2-3　　d) 4-6　　e) 7 or more

*Please indicate your level of agreement with the following statements:*

2. I am satisfied with the mentoring I have received in the past six months.

| Strongly Disagree | Disagree | Slightly Disagree | Neutral | Slightly Agree | Agree | Strongly Agree |
|---|---|---|---|---|---|---|
| 1 | 2 | 3 | 4 | 5 | 6 | 7 |

3. I would benefit from mentoring regarding clinical practice.

| Strongly Disagree | Disagree | Slightly Disagree | Neutral | Slightly Agree | Agree | Strongly Agree |
|---|---|---|---|---|---|---|
| 1 | 2 | 3 | 4 | 5 | 6 | 7 |

4. I would benefit from mentoring in research.

| Strongly Disagree | Disagree | Slightly Disagree | Neutral | Slightly Agree | Agree | Strongly Agree |
|---|---|---|---|---|---|---|
| 1 | 2 | 3 | 4 | 5 | 6 | 7 |

5. I would benefit from mentoring in academic related pursuits (advancement).

| Strongly Disagree | Disagree | Slightly Disagree | Neutral | Slightly Agree | Agree | Strongly Agree |
|---|---|---|---|---|---|---|
| 1 | 2 | 3 | 4 | 5 | 6 | 7 |

6. I would benefit from mentoring on topics of work-life integration.

| Strongly Disagree | Disagree | Slightly Disagree | Neutral | Slightly Agree | Agree | Strongly Agree |
|---|---|---|---|---|---|---|
| 1 | 2 | 3 | 4 | 5 | 6 | 7 |

7. I have been successful in finding mentors as I need them.

| Strongly Disagree | Disagree | Slightly Disagree | Neutral | Slightly Agree | Agree | Strongly Agree |
|---|---|---|---|---|---|---|
| 1 | 2 | 3 | 4 | 5 | 6 | 7 |

8. What best describes your position? (Please circle)

Resident　　Fellow　　Senior Associate Consultant　　Consultant

STUDY ID: (DOB: Month-Day)
Gender:
Age:
Academic Rank:

**Personal Goals (questions) for the Speed Mentoring Session:**

1.

2.

3.

# Mentoring Event: Evaluation (Mentee)

***Definition***: *A* ***mentoring relationship*** *is one that may vary along a continuum from informal/short-term to formal/long-term in which faculty with useful experience, knowledge, skills, and/or wisdom offers advice, information, guidance, support, or opportunity to another faculty member or student for that individual's professional development. (Berk RA, et al. Acad Med 80:66-71, 2005)*

*Please indicate your level of agreement with the following statements:*

1. My time today was well spent.

| Strongly Disagree | Disagree | Slightly Disagree | Neutral | Slightly Agree | Agree | Strongly Agree |
|---|---|---|---|---|---|---|
| 1 | 2 | 3 | 4 | 5 | 6 | 7 |

2. My discussions with mentors were stimulating.

| Strongly Disagree | Disagree | Slightly Disagree | Neutral | Slightly Agree | Agree | Strongly Agree |
|---|---|---|---|---|---|---|
| 1 | 2 | 3 | 4 | 5 | 6 | 7 |

3. The key question I asked of each mentor was adequately answered.

| Strongly Disagree | Disagree | Slightly Disagree | Neutral | Slightly Agree | Agree | Strongly Agree |
|---|---|---|---|---|---|---|
| 1 | 2 | 3 | 4 | 5 | 6 | 7 |

4. I am likely to pursue a mentoring relationship with one or more of the mentors here.

| Strongly Disagree | Disagree | Slightly Disagree | Neutral | Slightly Agree | Agree | Strongly Agree |
|---|---|---|---|---|---|---|
| 1 | 2 | 3 | 4 | 5 | 6 | 7 |

5. I would recommend to my colleagues that they participate in a similar event in the future.

| Strongly Disagree | Disagree | Slightly Disagree | Neutral | Slightly Agree | Agree | Strongly Agree |
|---|---|---|---|---|---|---|
| 1 | 2 | 3 | 4 | 5 | 6 | 7 |

6. Please indicate your overall rating of this event:

Complete waste of time 1 2 3 4 5 6 7 Outstanding

## Mentoring Event: Evaluation (Mentee, pg 2)

7. On average, how appropriate was the time allowed for each encounter?

a) Too short  b) Just right  c) Too long

8. What was your "key area" (ie: practice, research, QI, education, speaking engagement, advancement, work-life integration, other)?

9. What was good about this event?

10. What could we do to improve this event?

# Mentoring Event: Evaluation (Mentor)

***Definition**: A **mentoring relationship** is one that may vary along a continuum from informal/short-term to formal/long-term in which faculty with useful experience, knowledge, skills, and/or wisdom offers advice, information, guidance, support, or opportunity to another faculty member or student for that individual's professional development. (Berk RA, et al. Acad Med 80:66-71, 2005)*

*Please indicate your level of agreement with the following statements:*

1. My time today was well spent.

| Strongly Disagree | Disagree | Slightly Disagree | Neutral | Slightly Agree | Agree | Strongly Agree |
|---|---|---|---|---|---|---|
| 1 | 2 | 3 | 4 | 5 | 6 | 7 |

2. My discussions with mentees were stimulating.

| Strongly Disagree | Disagree | Slightly Disagree | Neutral | Slightly Agree | Agree | Strongly Agree |
|---|---|---|---|---|---|---|
| 1 | 2 | 3 | 4 | 5 | 6 | 7 |

3. I am likely to pursue a mentoring relationship with one or more of the mentees.

| Strongly Disagree | Disagree | Slightly Disagree | Neutral | Slightly Agree | Agree | Strongly Agree |
|---|---|---|---|---|---|---|
| 1 | 2 | 3 | 4 | 5 | 6 | 7 |

4. I would like to participate in a similar event in the future.

| Strongly Disagree | Disagree | Slightly Disagree | Neutral | Slightly Agree | Agree | Strongly Agree |
|---|---|---|---|---|---|---|
| 1 | 2 | 3 | 4 | 5 | 6 | 7 |

5. I would recommend to my colleagues that they participate in a similar event in the future.

| Strongly Disagree | Disagree | Slightly Disagree | Neutral | Slightly Agree | Agree | Strongly Agree |
|---|---|---|---|---|---|---|
| 1 | 2 | 3 | 4 | 5 | 6 | 7 |

6. Please indicate your overall rating of this event:

Complete waste of time 1 2 3 4 5 6 7 Outstanding

## Mentoring Event: Evaluation (Mentor, pg 2)

7. On average, how appropriate was the time allowed for each encounter?
   a) Too short  b) Just right  c) Too long

8. What was good about this event?

9. What could we do to improve this event?

Please complete the survey below.

Thank you!

**Please take a moment to respond to the following questions regarding The May 30th Speed Mentoring Event event, and mentoring in general.**

Date of Birth

______________________
(Month and Day)

How many mentors do you currently have?

- ◯ None
- ◯ 1
- ◯ 2-3
- ◯ 4-6
- ◯ 7 or more

**Please indicate your level of agreement with the following statements:**

| | Strongly Disagree | Disagree | Somewhat Disagree | Neutral | Somewhat agree | Agree | Strongly Agree |
|---|---|---|---|---|---|---|---|
| I am satisfied with the mentoring I have received since the Speed Mentoring Event. | ◯ | ◯ | ◯ | ◯ | ◯ | ◯ | ◯ |
| I would benefit from more intensive mentoring. | ◯ | ◯ | ◯ | ◯ | ◯ | ◯ | ◯ |
| I have been successful in finding mentors as I need them. | ◯ | ◯ | ◯ | ◯ | ◯ | ◯ | ◯ |

Have you had follow-up interactions with a mentor as a result of the Speed Mentoring sessions? Please elaborate on frequency and mode. (ie- face to face, email, phone, other).

______________________

Have you had advancement towards your goal as discussed at the event? I.e. project, research, schedule, etc.

______________________

Looking back, how useful was the mentoring event? What made it useful (or not useful)? What could we do to improve?

______________________
